# Standardized ileal digestibility of amino acids of protein sources associated with exogenous enzymes for broilers

**DOI:** 10.5713/ab.21.0416

**Published:** 2022-01-05

**Authors:** Bruno Duarte Alves Fortes, Heloisa Helena de Carvalho Mello, Marcos Barcellos Café, Emmanuel Arnhold, José Henrique Stringhini

**Affiliations:** 1 Federal Institute of Education, Science and Technology Goiano, Iporá, 76200-000, Brazil; 2 Department of Animal Science, Federal University of Goiás, Goiás, 74690-900, Brazil

**Keywords:** Enzyme Complex, Feedstuffs, Multivariate Analysis, Poultry

## Abstract

**Objective:**

Two experiments were conducted to evaluate the effect of enzyme complex (EC) on the standardized ileal digestibility (SID) of amino acids (AA) in corn gluten meal (60%) (CGM), soy protein concentrate (SPC), dried bovine plasma (DBP), and poultry offal meal (POM). Experiments I and II were conducted with broilers in the pre-starter (1 to 7 days of age) and starter (1 to 21 days of age) phases, respectively.

**Methods:**

The treatments consisted of a protein-free diet (PFD) containing feedstuffs either supplemented with EC (xylanase, amylase, and protease) or not. In Experiment I, a total of 360 one-day-old male Cobb-500 broiler chicks were randomly housed in 45 pens, resulting in five replicates with eight birds each, totalizing eight treatments and one PFD group. In Experiment II a total of 270 one-day-old male Cobb-500 broiler chicks were randomly housed in 45 pens, resulting in five replicates with six birds each, totalizing eight treatments and one PFD group. The PFD groups were used to assess the endogenous AA losses. The birds were slaughtered to collect the ileal content.

**Results:**

In the pre-starter phase, the SID of arginine, branched chain-aminoacids, glycine, serine, aspartate, and glutamic acid increased with EC addition. The EC improved the SID of arginine and glutamic acid of CGM; the SID of valine and cystine of SPC; the SID of leucine, glycine, and aspartate of POM and the SID of isoleucine of DBP. In the starter phase, the SID of isoleucine, phenylalanine and glycine increased in EC-supplemented diets. The EC improved the SID of isoleucine of DBP; the SID of phenylalanine of CGM and POM. The SID of AA of SPC was not influenced by the EC.

**Conclusion:**

The addition of an EC to broiler pre-starter and starter diets is efficient in increasing the SID of AA on SPC, POM, and DBP.

## INTRODUCTION

Alternative ingredients to corn and soybean meal have been used in broiler diets in the poultry industry; however, knowledge of the AA digestibility of these foods is a concern among nutritionists. Several factors are believed to affect AA digestibility, such as the bioassay and animal age [1], ingredient origin [2], the timing of planting and harvesting [3], a fermentation process of the ingredients [4] and exogenous enzyme complex (EC) [5,6].

The use of enzymes in their mono-component form or combined in blends is routine practice in poultry feeding. The efficacy of exogenous protease on AA digestibility in broiler diets that contain both xylanase and phytase was verified by [6]. Xylanase is effective to improve the ileal amino acid (AA) digestibility in broiler chickens [7]. Enzyme complexes such as xylanase, amylase and protease may be used in broilers’ diets, aiming to increase their performance and nutrient digestibility [8]. This better performance can be attributed to an enhancement of nutrient digestibility, including AA digestibility.

Different effects of the enzyme are observed according to the developmental stages of the birds. In young broilers, this is even more significant, since major differences in physiological development will impact their development throughout life [9,10]. In addition, it has been reported that bird age *per se* is a factor that affects AA digestibility of ingredients [1]. For this reason, the quality of ingredients and meeting the requirement of AAs for broilers in the pre-starter and starter phases of life is important to ensure their slaughter weight.

The main protein source for broilers is soybean meal. However, to reduce the diet costs, alternative by-products and an EC can be used. It was demonstrated that a multi-carbohydrase and phytase complex allows a saving in feed cost while guaranteeing the same performance of the broilers [11]. Corn gluten meal, soy protein concentrate (SPC), dried bovine plasma (DBP) and poultry offal meal (POM) provide a higher protein content and are commonly used in non-ruminant diets. The dietary AAs affect the body weight gain, the growth rate, the feed conversion ratio, the internal organ development [12], and the function of the gastrointestinal tract of broilers [13]. Therefore, the EC is used in diets to improve the digestibility of the AAs in by-products. Some authors concluded that feeding a combination of multi-carbohydrase and phytase results in higher standardized ileal digestibility (SID) of arginine, histidine, methionine, and threonine relative to a single activity in canola meal [14].

The present study was conducted to evaluate the effect of EC on the SID of AAs in CGM (60%), SPC, DBP, and POM to broiler pre-starter and starter diets.

## MATERIALS AND METHODS

### Animal care and study site

The experiments were conducted on the poultry facilities at the Department of Animal Science of the Federal University of Goiás (UFG), Brazil. Experimental protocols received approval from the Ethics Committee on Animal Use of UFG (case no. 066/12).

### Animals, experimental design

Two experiments were carried out to assess the SID of AA in CGM (60%), SPC, DBP, and POM with an EC or not, for broilers in the pre-starter (1 to 7 days of age, Experiment I) and starter phase (1 to 21 days of age, Experiment II).

In both experiments, a group of broilers were fed a pro tein-free diet (PFD) to assess the endogenous losses of AAs and calculate the SID of AAs.

In Experiment I, a total of 360 one-day-old male Cobb- 500 broiler chicks from a commercial hatchery were randomly housed in 45 pens, resulting in five replicates with eight birds each in the pre-starter phase, totalizing eight treatments and one PFD group.

In Experiment II a total of 270 one-day-old male Cobb- 500 broiler chicks from a commercial hatchery were randomly housed in 45 pens, resulting in five replicates with six birds each in the starter phase, totalizing eight treatments and one PFD group.

### Experimental diets

The experimental diets included four protein sources: CGM, SPC, DBP, and POM, which were each supplemented with EC or not, totalizing eight treatments.

The experimental diets were made with the inclusion of feedstuff to replace the cornstarch of PFD. CGM and SPC replaced 20% of the cornstarch in the diet. DBP and POM replaced 17% of the cornstarch in the diet (Table 1). The EC (Axtra) contained (per kg) 2,000 U xylanase, 200 U amylase and 4,000 U protease. Enzyme addition followed the manufacturer recommendations. The acid-insoluble ash Celite was included at 1% as an indigestible marker for calculating standardized AA digestibility [15]. The birds were allowed a period of five days of adaptation to the experimental diets. Water and feed were available *ad libitum* throughout the experimental period.

The dry matter (DM), crude protein (CP) contents and the total AA composition of the analyzed feedstuffs are described in Table 2.

### Broiler management

From one to seven (Experiment I) and one to 21 days of age (Experiment II), the chicks were raised in five broiler battery galvanized steel cages equipped with trough-type feeders and drinkers. Each battery contained five floors with 0.33×0.50 m divisions. To heat the birds, 40-watt incandescent lamps were used for each battery floor until the birds completed 7 and 14 days of age, in Experiments I and II, respectively. The broilers were managed according to the management guidelines of the line. Ambient temperature and relative humidity were recorded daily, and adequate curtain management was adopted.

### Ileal content sample collection

At seven (Experiment I) and 21 (Experiment II) days of age, all birds from all replicates were slaughtered by cervical dislocation and immediately dissected at 5 cm from the ileocecal-colic junction to 40 cm towards the jejunum for ileal content collection and determination of endogenous AA losses, following Sakomura and Rostagno [15] recommendations.

The ileal content was packed in plastic bags, labelled, and stored in a freezer at −16°C. Samples of ingredients, diets and ileal contents were vacuum-freeze-dried at a temperature of −40°C for 72 hours and sent to the Animal Nutrition Laboratory at EVZ/UFG for processing and analysis of the DM and CP contents of feedstuffs and ileal content. The DM content was determined by drying in an oven (105°C), the CP by the micro-Kjeldahl method (nitrogen distiller Tecnal TE-0364; Tecnal Industry Trade Import and Export of Scientific Laboratory Equipment, Piracicaba, SP, Brazil). The analysis was determined according to the procedures described by Silva and Queiroz [16]. To determine the total AA content, high performance/pressure liquid chromatography was performed at the Laboratory of Evonik Industries (Hanau, Germany).

### Calculations

The SID of AAs was calculated according to the proposal equations from Sakomura and Rostagno [15], as follow:


AID (%)=[(Aadiet [DM,%]/AIAdiet [%])-(AA ileal digesta [%]/AIA ileal digesta [%])]×100/AA diet (DM,%)/AIAdiet (%)

EAA (g/kg DM)=AA digesta (g/kg DM)×[AIA diet (g/kg DM)/AIA digesta (g/kg DM)]

SID (%)=AID (%)+[EAA (g/kg DM)/AA digt (g/kg DM)]×100

in which: AID, apparent ileal digestibility of the AA; AIA, acid-insoluble ash; SID, standardized ileal digestibility of AA; DM, dry matter; EAA, basal endogenous AA loss.

The standardized AA content of feedstuffs was calculated according to the total AA content and the SID and presented as a fed basis.

### Statistical analysis

The data were subjected to analysis of variance, and to mean comparison by the Scott Knott test at the 5% significance level, using the R statistical program, R Core Team 2021. The statistical model included the fixed effects of feedstuffs and EC supplementation, and their interaction effect, and the random effects of the experimental unit. Data were analyzed within each feedstuff by analysis of variance to test the effects of EC within each ingredient. A multivariate analysis of principal components was performed to assess the interrelationships between variables and treatments.

## RESULTS

The SID of AA in CGM (60%), SPC, DBP, and POM, determined with broilers at seven days of age, are described in Table 3. There were verified differences in SID of all AA according to the feedstuffs. The SPC presents a higher SID of AA. However, the SID of lysine, methionine, histidine, alanine, and aspartate were similar between the CPS and the DPB. The CGM presents the lowest SID of methionine, methionine plus cystine, threonine, arginine, histidine, leucine, phenylalanine, serine, alanine, and glutamic acid. The SID of arginine, branched chain-aminoacids, glycine, serine, aspartate, and glutamic acid increased with EC addition (Table 3).

The SID of AA within each feedstuff with or without EC in pre-starter diets is shown in Table 4. The SID of arginine and glutamic acid of CGM increased with EC addition. It was observed that the SID of valine and cystine of SPC were higher with EC inclusion. The POM presented a higher SID of leucine, glycine, and aspartate when the EC was used. The SID of isoleucine of DBP increased with EC inclusion.

The SID of AA in feedstuffs determined with broilers at 21 days of age is described in Table 5. It was observed, unlike what occurred in seven days old, that the SID of some AA of SPC, DBP and CGM were similar (methionine, cystine, methionine plus cystine, threonine, valine, serine, proline, glutamic acid), indicating better utilization of AAs of alternative feedstuffs with increased age. Furthermore, the lowest SID of indispensable AA in POM was verified. The SID of isoleucine, phenylalanine and glycine increased with EC addition in the broiler starter diet (Table 5).

The SID of AAs (%) of each feedstuff with or without EC on starter diets of broilers is described in Table 6. The EC improved the SID of phenylalanine of CGM. The SID of AA of SPC was not influenced by the EC in diets. The DBP presents a higher SID of isoleucine when the EC was used.

The standardized AA contents of the analyzed feedstuffs are described in Table 7. Although the feedstuffs studied are protein sources, the contents of AA in each presented great variation.

**Figure 1:**
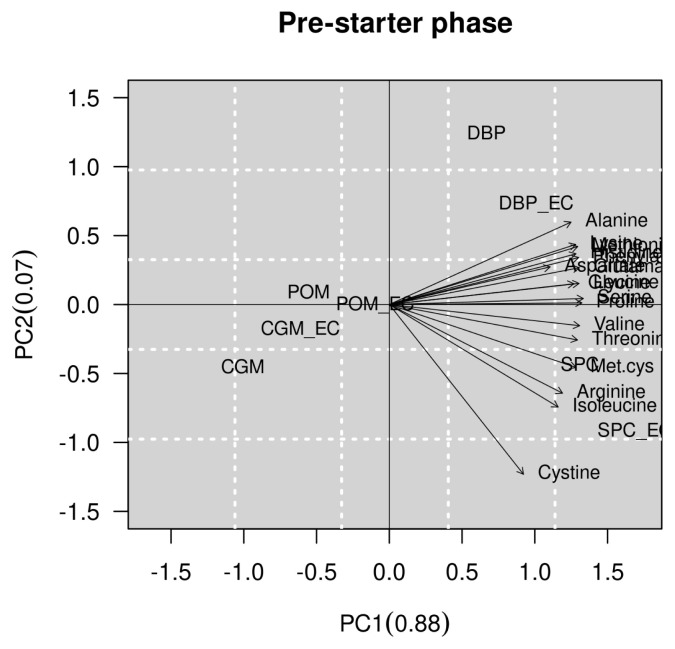
2D plot generated by principal component analysis. The graph shows a separation between the feedstuffs with or without the enzyme complex and their relationship to the standardized ileal digestibility (SID) of amino acids (AA), based on multivariate analysis in pre-starter phase. CGM, corn gluten meal; CGM_EC, corn gluten meal with enzyme complex; SPC, soy protein concentrate; SPC_EC, soy protein concentrate with enzyme complex; POM, poultry offal meal; POM_EC, poultry offal meal with enzyme complex; DBP, dried bovine plasma; DBP_EC, dried bovine plasma with enzyme complex.

**Figure 2:**
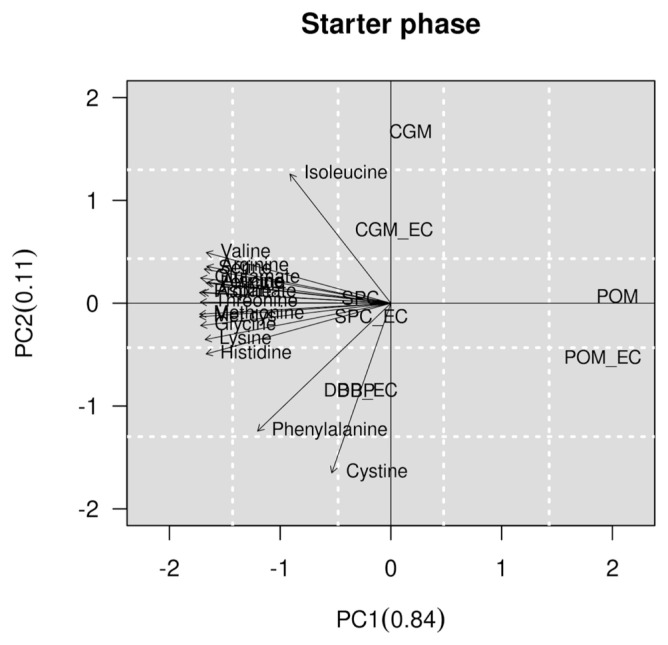
2D plot generated by principal component analysis. The graph shows a separation between the feedstuffs with or without the enzyme complex and their relationship to standardized ileal digestibility (SID) of amino acids (AA), based on multivariate analysis in starter phase. CGM, corn gluten meal; CGM_EC, corn gluten meal with enzyme complex; SPC, soy protein concentrate; SPC_EC, soy protein concentrate with enzyme complex; POM, poultry offal meal; POM_EC, poultry offal meal with enzyme complex; DBP, dried bovine plasma; DBP_EC, dried bovine plasma with enzyme complex.

The results of multivariate analysis are presented in Figure 1 and 2 of the pre-starter and starter phases, respectively. It was observed that the SID of AA of SPC with or without an EC was higher than the SID of AA of CGM, DBP and POM at the pre-starter and starter phases. In the pre-starter phase, we verified that the CGM shows the lowest SID of AA, however, at the starter phase the POM shows the lowest SID of AA (Figure 1). There was no negative correlation between the SID of AA to feedstuffs studied, but there was a clear positive correlation between the SID of some AA studied, such as proline and serine in the pre-starter phase. At the starter phase, the SID of phenylalanine, isoleucine and cystine showed no correlation (Figure 2).

## DISCUSSION

The aim of this study was to evaluate the effect of EC on the SID of AA of four protein sources in pre-starter and starter broilers’ diets. Knowledge of the AA digestibility of feed ingredients is of great importance, especially when aiming to use an unconventional feedstuff in broiler diets. In the present study, it was verified that in the pre-starter phase the SID of arginine, branched chain-aminoacids, glycine, serine, aspartate, and glutamic acid was increased in EC-supplemented diets. In contrast, at the starter phase, only the SID of isoleucine, phenylalanine and glycine increased with EC addition to the diet. This improvement occurred independently of the feedstuff studied. In addition, the experiments showed that the SID of AA is different according to the feedstuffs.

On average, SPC showed the highest SID of all the feed stuffs tested in this study, which makes it an excellent protein source for birds in the pre-starter phase. The high digestibility of AA in pre-starter diets is important, since it has been related that dietary AA affects the body weight gain, the growth rate, the feed conversion ratio, the internal organ development of broilers [12] and the improved function of the gastrointestinal tract of broilers [13]. Some factors influence the AA digestibility in broilers. [17] verified that the defatting process of insect meal increased glutamic acid, proline, and serine digestibility. Yaghobfar [1] verified that the AA digestibility of soybean, sunflower and canola meals was lower in broilers than in intact and caecectomized cockerels, indicating that age affects the use of AA by broilers.

The SID of lysine, methionine, histidine, alanine, and as partate were similar between the CPS and the DPB, indicating that DPB is a great alternative animal byproduct to use in pre-starter broilers’ diets. Furthermore, the SID of isoleucine of DBP increased with EC inclusion. Lysine, methionine, and threonine are the first three limiting AA for broilers fed a corn-soybean meal diet and are present in great quantities in SPC and DPB. Therefore, [18] related that the spray-dried plasma improves early intestinal health and supports an efficient immune system response both locally at the intestine and systemically, thereby benefiting growth, feed efficiency, and survival of broilers.

The CGM presented the lowest SID of indispensable AA in pre-stater diets. However, the addition of EC was able to increase the SID of arginine and glutamic acid in the broilers fed the diet containing CGM. The CGM showed the lowest digestibility coefficients among the plant-based products, which may be due to its high fibre content, which reduces the AA digestibility.

At the pre-starter phase, the SID of arginine, branched chain-aminoacids, glycine, serine, aspartate, and glutamic acid increased in EC-supplemented diets. These AA are classified as polar and non-polar, indicating that the EC did not improve the digestibility of a specific group of AAs. The SID of these AA was increased up to 2.41% in mean, with the EC inclusion. The results agree with [6], verifying that the exogenous protease addition resulted in an increase in ileal AA digestibility of over 2.5%. There are many mechanisms by which the enzyme improves the SID of AA. The EC reduces the viscosity of digesta [19–21], increases digesta retention time in the ileum [22], facilitating the digestion of nutrients by the digestive enzymes. Furthermore, it has been shown that the phytase plus xylanase combination increased sodium digestibility [22]. Since AA absorption is sodium-dependent, the increase of sodium digestibility could result in an enhanced SID of AA. In addition to these factors, the addition of exogenous protease to the diet increases the jejunal expression of genes responsible for peptide transport [6], resulting in better utilization of some AA. Cowieson et al [23] reported that exogenous protease may influence digestive dynamics through altered secretion of intestinal mucin, improved tight junction integrity and changed emphasis on AA transport.

The SID of AA within each feedstuff with or without EC was studied in the pre-starter phase. In the SPC, the SID were significantly higher for valine and cystine in the EC-supplemented diets. It was verified that all the AA present SID up to 90% in SPC. The inherent high digestibility of AA of SPC could contribute to a lack of effect of EC in enhancing the SID of other AA.

The SID of leucine, glycine and aspartate on POM was improved when the EC was used. The improvement of glycine digestibility in the pre-starter diet is important. Dietary glycine may need to be considered as a limiting nutrient in early nutrition, especially if the CP is low, and only vegetable ingredients are being used [24].

At the starter phase, it was verified that the SID of some AA of SPC, DBP, and CGM was similar, indicating better utilization of AAs of alternative feedstuffs with the increase of age. In this context, it is important to evaluate differences in AA digestibility in broiler chicks in the first week of life, given the limitations that occur in the digestive processes that affect nutritional utilization at that stage [25,26]. Because AA digestibility in birds is determined in the third week of life, differences in physiological maturity, especially in terms of intestinal functions and enzyme action, are greater than in the pre-starter phase, which can affect nutrient utilization efficiency and body development in these animals.

At the starter phase, only the SID of isoleucine, phenyl alanine and glycine increased with EC addition in the diet (average of increase of 2.33%). At the starter phase, the digestive system of the broiler is completely developed, and age could influence the SID of AA. Szczurek et al [27] verified that the SID value of AA in wheat was not influenced by the age of broilers, but the SID of most AA in triticale, and all the AA in barley, were higher in 28-day-old chickens compared with 14-day-olds. The results suggested that the SID coefficients of AA are influenced by the age of birds in a feedstuff-dependent way. In addition to the age of birds, other factors can affect the efficacy of exogenous enzymes and the AA digestibility. The dietary levels of available phosphorus and calcium can influence the efficacy of the combination of a multi-carbohydrase and phytase complex, on the digestibility of AAs of broilers [28].

The standardized AA contents of the feedstuffs were cal culated from the total AA content and the SID. Although the feedstuffs studied are protein sources, the contents of AA in each presented great variation. The digestible AA content of SPC was, on average, lower than those observed by [29], probably due to the lower protein levels found in this feedstuff in the present experiment. In fact, differences in nutritive value do exist between SBM from different origins in terms of nutrient contents, apparent metabolizable energy, and digestible AA [2].

The POM showed the greatest variation in digestible AA content between the feedstuffs of animal origin. This result can be attributed to the broad variations in its composition, which also explains the divergent AA compositions between different studies. The observed differences in AA content can be attributed to a lack of standardization in the processing of the evaluated feedstuffs. The processing of a feedstuff greatly influences the digestibility of AA. Overprocessing, for instance, can lead to a deficiency in sulfur-containing AAs, especially cystine, which is converted to lanthionine, which in turn has low nutritional value. On the other hand, insufficient processing may result in incomplete hydrolysis, which translates into lower nutrient digestibility [30]. The reduced digestibility of lysine may be attributed to the formation of Maillard reaction products during thermal processing.

In summary, we verified that the EC effectively improved the SID of AA of feedstuffs, resulting in the better utilization of some AA by birds. The addition of EC increased AA digestibility by an average of 2.41% and 2.33%, respectively, at the pre-starter and starter phases.

## CONCLUSION

The addition of an EC to broiler pre-starter and starter diets is efficient in increasing the SID of AA on SPC, POM and DBP.

## Figures and Tables

**Table 1:** Ingredient composition of the experimental diets fed to broilers, as fed-basis (%) (Experiment I and II)

Ingredient	PFD	CGM	CGM+EC	SPC	SPC+EC	POM	POM+EC	DBP	DBP+EC
Starch	81.0	61.0	60.95	61.0	60.95	64.0	63.95	64.0	63.95
Corn gluten meal, 60%	-	20.0	20.00	-	-	-	-	-	-
Soy protein concentrate	-	-	-	20.0	20.0	-	-	-	-
Offal meal	-	-	-	-	-	17.0	17.0	-	-
Dried bovine plasma	-	-	-	-	-	-	-	17.0	17.0
Soybean oil	4.00	4.00	4.00	4.00	4.00	4.00	4.00	4.00	4.00
Dicalcium phosphate	2.65	2.65	2.65	2.65	2.65	2.65	2.65	2.65	2.65
Limestone	0.70	0.70	0.70	0.70	0.70	0.70	0.70	0.70	0.70
Common salt	0.60	0.60	0.60	0.60	0.60	0.60	0.60	0.60	0.60
Sugar	5.00	5.00	5.00	5.00	5.00	5.00	5.00	5.00	5.00
Vitamin-mineral supplement^1)^	0.04	0.04	0.04	0.04	0.04	0.04	0.04	0.04	0.04
Enzyme complex^2)^	-	-	0.05	-	0.05	-	0.05	-	0.05
Antioxidant^3)^	0.01	0.01	0.01	0.01	0.01	0.01	0.01	0.01	0.01
Inert^4)^	5.00	5.00	5.00	5.00	5.00	5.00	5.00	5.00	5.00
Marker^5)^	1.00	1.00	1.00	1.00	1.00	1.00	1.00	1.00	1.00
Total	100.0	100.0	100.0	100.0	100.0	100.0	100.0	100.0	100.0

PFD, diet protein free; CGM, corn gluten meal; EC, enzyme complex; SPC, soy protein concentrate; POM, poultry offal meal; DBP, dried bovine plasma.

1) Provides per kilogram of product: folic acid 200 mg, pantothenic acid 3,120 mg, biotin 10 mg, copper 1,997 mg, choline 78.10 g, enramycin 2,000 mg, iron 11.25 g, iodine 187.47 mg, maduramicin 937.50 mg, manganese 18.74 g, niacin 8,400 mg, nicarbazin 10 g, selenium 75 mg, vitamin A 1,680,000 IU, vitamin B_1_ 436.50 mg, vitamin B_12_ 2,400 mcg, vitamin B_2_ 1,200 mg, vitamin B_6_ 624 mg, vitamin D_3_ 400,000 IU, vitamin E 3,500 IU, vitamin K_3_ 360 mg, zinc 17.50 g.

2) Axtra®.

3) BHT (butylated hydroxytoluene).

4) Rice husk.

5) Celite (ash insoluble in acid).

**Table 2:** Dry matter, crude protein contents and total amino acid composition of different feedstuffs for broiler, expressed in percentage (as-fed basis)^1)^

Items	CGM	SPC	POM	DBP
Dry matter	91.62	94.03	95.01	92.08
Crude protein	62.09	62.36	63.73	70.30
Indispensable AA				
Lysine	1.01	3.81	4.14	6.86
Methionine	1.47	0.84	1.39	0.81
Methionine+cystine	2.58	1.73	2.05	3.68
Threonine	2.07	2.52	2.51	4.95
Arginine	1.97	4.55	4.16	4.01
Histidine	1.33	1.60	1.06	2.88
Valine	2.83	2.80	2.93	4.96
Isoleucine	2.51	2.74	2.42	2.35
Leucine	10.23	4.85	4.48	7.21
Phenylalanine	3.90	3.20	2.51	4.41
Glycine	1.49	2.64	4.74	2.98
Dispensable AA				
Cystine	1.10	0.89	0.67	2.17
Alanine	5.51	2.72	3.34	4.02
Aspartic acid	3.77	7.25	4.49	8.12
Glutamic acid	13.59	11.28	7.02	11.79
Serine	3.12	3.34	4.60	4.98
Proline	1.56	3.04	4.65	4.11

1) Aminogram developed by Evonik Industries AG Feed Additives/Animal Nutrition Services.

CGM, corn gluten meal; SPC, soy protein concentrate; POM, poultry offal meal; DBP, dried bovine plasma; AA, amino acid.

**Table 3:** Standardized ileal digestibility of amino acids (%) of feedstuffs determined in seven-day-old broilers

Items	Lys	Met	Cys	M+C	Thr	Arg	His	Val	Iso	Leu	Phe	Gly	Ser	Pro	Ala	Asp	Glu
Feedstuffs (F)																	
CGM (60%)	81.62^b^	78.06^c^	79.60^b^	77.74^c^	77.79^d^	82.04^c^	73.44^c^	78.96^c^	79.38^b^	75.95^d^	74.16^c^	76.44^c^	74.48^d^	73.00^c^	73.66^c^	74.46^b^	73.03^d^
SPC	95.04^a^	97.57^a^	91.87^a^	97.35^a^	96.88^a^	98.33^a^	96.11^a^	93.98^a^	94.41^a^	95.11^a^	98.27^a^	93.36^a^	98.47^a^	97.73^a^	93.47^a^	91.67^a^	96.84^a^
POM	85.26^b^	84.86^b^	78.90^b^	80.33^c^	84.47^c^	87.01^b^	83.71^b^	78.96^c^	79.71^b^	84.08^c^	83.58^b^	74.04^d^	78.42^c^	74.53^c^	82.01^b^	62.82^c^	82.51^c^
DBP	94.63^a^	96.91^a^	78.77^b^	86.73^b^	88.73^b^	87.56^b^	94.54^a^	88.35^b^	83.98^b^	91.18^b^	95.87^a^	90.42^b^	91.92^b^	90.85^b^	95.37^a^	92.40^a^	93.21^b^
SEM	1.50	0.92	1.78	1.12	1.00	0.64	1.24	0.68	1.63	0.60	1.10	0.71	0.76	1.20	1.11	0.54	0.74
Enzyme complex (EC)																	
−	87.94	88.57	80.56	85.01	86.17	87.83^b^	85.83	84.24^b^	82.60	85.59^b^	86.82	82.65^b^	84.89^b^	82.70	85.09	79.13^b^	85.08^b^
+	90.33	90.13	84.01	86.06	87.77	89.64^a^	88.07	85.88^a^	86.14	87.57^a^	89.12	84.47^a^	86.75^a^	85.36	87.17	81.55^a^	87.72^a^
SEM	1.06	0.65	1.25	0.79	0.71	0.45	0.87	0.48	1.15	0.84	0.77	0.50	0.54	0.84	0.78	0.38	0.52
p-value																	
F	<0.001	<0.001	0.002	<0.001	<0.001	<0.001	<0.001	<0.001	<0.001	<0.001	<0.001	<0.001	<0.001	<0.001	<0.001	<0.001	<0.001
EC	0.1502	0.1331	0.0888	0.381	0.15	0.0223	0.1082	0.044	0.0624	0.0109	0.0709	0.0337	0.0415	0.0581	0.0971	0.0022	0.0078
F×EC	0.5725	0.9601	0.2853	0.9226	0.7057	0.4947	0.9824	0.4705	0.2612	0.585	0.7477	0.5042	0.5221	0.8378	0.7576	0.0067	0.3278

M+C, methionine+cystine; CGM, corn gluten meal; SPC, soy protein concentrate; POM, poultry offal meal; DBP, dried bovine plasma; SEM, standard error of the means; (−) without EC; (+) with EC.

a–d Means within a column-subgroup with no common superscript letters are significantly different at p<0.05 by Scott Knott test.

**Table 4:** Standardized ileal digestibility of amino acids (%) of feedstuffs with or without enzyme complex in seven-day-old broilers

Items	Corn gluten meal (60%)				Soy protein concentrate				Poultry offal meal				Dried bovine plasma			
			
	−	+	SEM	p-value	−	+	SEM	p-value	−	+	SEM	p-value	−	+	SEM	p-value
Indispensable AA																
Lys	78.64	84.59	2.12	0.082	94.62	95.47	2.12	0.782	84.06	86.46	2.12	0.447	94.45	94.80	2.12	0.91
Met	77.00	79.12	1.31	0.285	96.70	98.45	1.31	0.374	84.07	85.65	1.31	0.419	96.53	97.29	1.31	0.693
M+C	77.73	77.76	1.59	0.989	96.57	98.13	1.59	0.508	80.03	80.63	1.59	0.796	85.73	87.72	1.59	0.403
Thr	77.71	77.78	1.42	0.934	95.60	98.16	1.42	0.239	82.97	85.98	1.42	0.172	88.40	89.07	1.42	0.748
Arg	80.30^b^	83.79^a^	0.90	0.026	97.94	98.73	0.90	0.552	86.10	87.93	0.90	0.191	87.00	88.13	0.90	0.402
His	72.51	74.37	1.75	0.476	94.67	97.56	1.75	0.277	82.44	84.89	1.75	0.334	93.71	95.38	1.75	0.518
Val	78.16	79.76	0.96	0.276	92.34^b^	95.62^a^	0.96	0.043	78.16	79.77	0.96	0.273	88.32	88.37	0.96	0.971
Iso	78.65	80.10	2.31	0.669	93.92	94.90	2.31	0.772	78.72	80.71	2.31	0.559	79.10^b^	88.86^a^	2.31	0.017
Leu	75.31	76.59	0.84	0.317	94.27	95.96	0.84	0.198	82.36^b^	85.81^a^	0.84	0.0207	90.43	91.93	0.84	0.246
Phe	72.44	75.89	1.55	0.156	97.14	99.39	1.55	0.337	82.00	85.17	1.55	0.1882	95.72	96.03	1.55	0.891
Gly	75.28	77.60	1.00	0.142	93.07	93.66	1.00	0.689	72.32^b^	75.76^a^	1.00	0.042	89.95	90.89	1.00	0.529
Dispensable AA																
Cys	80.47	78.74	2.52	0.641	87.38^b^	96.35^a^	2.52	0.035	77.22	80.57	2.52	0.3744	77.17	80.37	2.52	0.395
Ala	71.97	75.36	1.57	0.166	92.08	94.87	1.57	0.245	81.00	83.03	1.57	0.3888	95.30	95.45	1.57	0.946
Asp	74.12	74.81	0.77	0.547	91.45	91.89	0.77	0.700	59.22^b^	66.43^a^	0.77	<0.001	91.74	93.08	0.77	0.255
Glu	70.56^b^	75.51^a^	1.05	0.010	96.05	97.63	1.05	0.322	81.03	84.00	1.05	0.0831	92.69	93.75	1.05	0.497
Ser	73.24	75.72	1.08	0.143	98.31	98.63	1.08	0.842	76.71	80.14	1.08	0.055	91.32	92.51	1.08	0.459
Pro	71.45	74.56	1.69	0.231	97.34	98.12	1.69	0.753	72.72	76.35	1.69	0.1697	89.30	92.40	1.69	0.232

SEM, standard error of the means; AA, amino acids; M+C, methionine+cystine.

a,b Means within a feedstuff group with no common superscript letters are significantly different at p<0.05 by Scott Knott test.

**Table 5:** Standardized ileal digestibility of amino acids (%) of feedstuffs determined in 21-day-old broilers

Items	Lys	Met	Cys	M+C	Thr	Arg	His	Val	Iso	Leu	Phe	Gly	Ser	Pro	Ala	Asp	Glu
Feedstuffs (F)																	
CGM (60%)	91.51^c^	94.60^b^	86.71	92.77^a^	93.60^a^	94.75^b^	89.21^b^	94.51^a^	93.80^a^	95.80^a^	83.74 b	88.51^b^	95.14^a^	92.57^a^	94.86^a^	89.88^b^	94.43^a^
SPC	94.29^b^	98.28^a^	91.40	95.11^a^	94.71^a^	97.11^a^	94.06^a^	93.92^a^	93.62^a^	93.24^b^	98.42 a	91.31^a^	96.17^a^	95.61^a^	91.88^b^	88.81^b^	95.11^a^
POM	81.48^d^	82.56^c^	89.41	83.27^b^	82.03^b^	84.12^c^	80.57^c^	80.43^b^	79.77^b^	81.74^c^	83.73 b	77.93^c^	82.56^b^	75.33^b^	80.76^c^	64.81^c^	79.21^b^
DBP	97.88^a^	98.38^a^	92.91	95.89^a^	96.54^a^	93.70^b^	96.07^a^	92.39^a^	80.53^b^	96.89^a^	97.61 a	93.01^a^	94.40^a^	94.67^a^	96.01^a^	94.44^a^	94.83^a^
SEM	0.41	0.47	2.34	1.20	1.88	0.56	0.99	1.09	0.71	0.66	0.88	0.59	1.01	1.00	0.67	0.42	0.80
Enzyme complex (EC)																	
−	91.09	93.00	88.13	91.26	91.65	92.08	89.49	89.79	86.05^b^	91.36	89.45^b^	86.91^b^	91.17	88.58	90.18	84.27	90.49
+	91.48	93.91	92.08	92.26	91.80	92.76	90.46	90.83	87.80^a^	92.47	92.30^a^	88.46^a^	92.97	90.51	91.57	84.70	91.30
SEM	0.29	0.33	1.65	0.85	1.33	0.39	0.70	0.77	0.50	0.46	0.62	0.42	0.71	0.70	0.47	0.29	0.57
p-value																	
F	<0.001	<0.001	0.3366	<0.001	0.0024	<0.001	<0.001	<0.001	<0.001	<0.001	<0.001	<0.001	<0.001	<0.001	<0.001	<0.001	<0.001
EC	0.3668	0.092	0.1301	0.432	0.9386	0.2617	0.3573	0.3713	0.04	0.134	0.0121	0.0311	0.112	0.0903	0.0733	0.3455	0.3473
F×EC	0.5727	0.6154	0.7188	0.9688	0.9984	0.5755	0.8045	0.8951	0.1352	0.6389	0.0743	0.9904	0.5991	0.7419	0.9579	0.1436	0.5604

M+C, methionine+cystine;CGM, corn gluten meal; SPC, soy protein concentrate; POM, poultry offal meal; DBP, dried bovine plasma; SEM, standard error of the means; (−) without EC; (+) with EC.

a–d Means within a column-subgroup with no common superscript letters are significantly different at p<0.05 by Scott Knott test.

**Table 6:** Standardized ileal digestibility of amino acids (%) of feedstuffs with or without enzyme complex in 21-day-old broilers

Items	Corn gluten meal (60%)				Soy protein concentrate				Poultry offal meal				Dried bovine plasma			
			
	−	+	SEM	p-value	−	+	SEM	p-value	−	+	SEM	p-value	−	+	SEM	p-value
Indispensable AA																
Lys	91.20	91.82	0.58	0.471	94.11	94.48	0.58	0.664	80.93	82.04	0.58	0.211	98.16	97.62	0.58	0.532
Met	93.68	95.53	0.67	0.087	97.95	98.62	0.67	0.501	82.03	83.10	0.67	0.293	98.36	98.41	0.67	0.959
M+C	91.81	93.73	1.70	0.484	94.61	95.62	1.70	0.686	82.89	83.66	1.70	0.757	95.75	96.04	1.70	0.907
Thr	93.24	93.97	2.66	0.851	94.76	94.66	2.66	0.979	82.06	82.02	2.66	0.990	96.54	96.56	2.66	0.996
Arg	94.27	95.24	0.79	0.411	97.19	97.04	0.79	0.893	83.19	85.06	0.79	0.132	93.70	93.72	0.79	0.989
His	87.96	90.47	1.40	0.243	94.10	94.03	1.40	0.974	80.00	81.16	1.40	0.577	95.93	96.23	1.40	0.885
Val	93.32	95.72	1.54	0.304	93.50	94.35	1.54	0.707	80.04	80.83	1.54	0.725	92.35	92.44	1.54	0.966
Iso	92.21	95.39	1.01	0.057	94.13	93.12	1.01	0.503	79.33	80.21	1.01	0.556	78.55^b^	82.52^a^	1.01	0.024
Leu	94.86	96.75	0.93	0.192	92.68	93.81	0.93	0.419	80.87	82.62	0.93	0.224	97.07	96.73	0.93	0.804
Phe	80.90^b^	86.57^a^	1.25	0.012	98.33	98.53	1.25	0.914	80.90^b^	86.57^a^	1.25	0.012	97.48	97.75	1.25	0.882
Gly	87.83	89.19	0.84	0.286	90.55	92.07	0.84	0.237	77.00	78.87	0.84	0.154	92.28	93.74	0.84	0.255
Dispensable AA																
Cys	82.64	90.80	3.31	0.119	90.75	92.06	3.31	0.786	87.11	91.73	3.31	0.352	92.06	93.77	3.31	0.724
Ala	94.20	95.53	0.95	0.351	91.29	92.48	0.95	0.403	79.77	81.77	0.95	0.176	95.50	96.53	0.95	0.465
Asp	89.11	90.66	0.59	0.104	89.48	88.15	0.59	0.154	64.72	64.91	0.59	0.827	93.80	95.09	0.59	0.167
Glu	93.67	95.20	1.14	0.370	95.53	94.71	1.14	0.622	78.07	80.36	1.14	0.193	94.73	94.95	1.14	0.892
Ser	94.92	95.38	1.43	0.825	96.00	96.35	1.43	0.866	80.79	84.35	1.43	0.116	92.98	95.84	1.43	0.195
Pro	91.79	93.36	1.41	0.457	95.45	95.77	1.41	0.877	73.65	77.03	1.41	0.130	93.45	95.91	1.41	0.254

SEM, standard error of the means; AA, amino acids; M+C, methionine+cystine.

a,b Means within a feedstuff group with no common superscript letters are significantly different at p<0.05 by Scott Knott test.

**Table 7:** Standardized digestible amino acid contents of feedstuffs (%) (as-fed basis)^1)^

Items	CGM	SPC	POM	DBP
Dry matter	91.62	94.03	95.01	92.08
Crude protein	62.09	62.36	63.73	70.30
Lysine	0.92	3.43	3.31	5.95
Methionine	1.35	0.77	1.15	0.66
Methionine+cystine	2.33	1.49	1.54	3.26
Threonine	1.94	2.36	1.93	4.28
Arginine	1.79	4.24	3.83	3.33
Histidine	1.21	1.47	0.93	2.35
Valine	2.57	2.46	2.32	4.40
Isoleucine	2.26	2.44	1.94	1.99
Leucine	9.31	4.31	3.63	6.20
Phenylalanine	3.51	2.85	2.11	3.71
Cystine	1.02	0.79	0.46	1.77

1) Aminogram developed by Evonik Industries AG Feed Additives/Animal Nutrition Services.

CGM, corn gluten meal; SPC, soy protein concentrate; POM, poultry offal meal; DBP, dried bovine plasma;
